# CRAFT: a web-integrated cavity prediction tool based on flow transfer algorithm

**DOI:** 10.1186/s13321-024-00803-6

**Published:** 2024-01-30

**Authors:** Anuj Gahlawat, Anjali Singh, Hardeep Sandhu, Prabha Garg

**Affiliations:** 1 Department of Pharmacoinformatics, National Institute of Pharmaceutical Education and Research (NIPER), Sector 67, S.A.S. Nagar, 160062 Punjab India; 2 Department of Computer Science, Kurukshetra University, Kurukshetra, Haryana India

**Keywords:** Cavity prediction, Flow transfer algorithm, CRAFT, Delaunay triangulation, Maximum circle radius (MCR), Protein cavities, Geometrical methods

## Abstract

**Abstract:**

Numerous computational methods, including evolutionary-based, energy-based, and geometrical-based methods, are utilized to identify cavities inside proteins. Cavity information aids protein function annotation, drug design, poly-pharmacology, and allosteric site investigation. This article introduces “flow transfer algorithm” for rapid and effective identification of diverse protein cavities through multidimensional cavity scan. Initially, it identifies delimiter and susceptible tetrahedra to establish boundary regions and provide seed tetrahedra. Seed tetrahedron faces are precisely scanned using the maximum circle radius to transfer seed flow to neighboring tetrahedra. Seed flow continues until terminated by boundaries or forbidden faces, where a face is forbidden if the estimated maximum circle radius is less or equal to the user-defined maximum circle radius. After a seed scanning, tetrahedra involved in the flow are clustered to locate the cavity. The CRAFT web interface integrates this algorithm for protein cavity identification with enhanced user control. It supports proteins with cofactors, hydrogens, and ligands and provides comprehensive features such as 3D visualization, cavity physicochemical properties, percentage contribution graphs, and highlighted residues for each cavity. CRAFT can be accessed through its web interface at http://pitools.niper.ac.in/CRAFT, complemented by the command version available at https://github.com/PGlab-NIPER/CRAFT/.

**Scientific contribution:**

Flow transfer algorithm is a novel geometric approach for accurate and reliable prediction of diverse protein cavities. This algorithm employs a distinct concept involving maximum circle radius within the 3D Delaunay triangulation to address diverse van der Waals radii while existing methods overlook atom specific van der Waals radii or rely on complex weighted geometric techniques.

**Supplementary Information:**

The online version contains supplementary material available at 10.1186/s13321-024-00803-6.

## Introduction

Structural biology plays a crucial role in understanding numerous aspects of proteins involving functions, interactions, signaling, evolutionary origin, and altered conditions. Pharmaceutical sciences and biotechnology have been utilizing various structure-based methods for the classification and functional annotation of biological entities [1, 2]. Currently, structure-based methods are incorporated into the standard pipeline of the drug development process to predict protein‒ligand complexes, their stability, and binding affinity [3]. Protein functions are primarily carried out by molecular recognition of their interactive interfaces by small or large molecules. Precisely, these interactive surfaces are cavities whose chemical makeup determines with which molecules a protein can interact. In proteins, cavities can exist in various forms, such as voids, clefts, pockets, and channels [4, 5]. Cavities are helpful in maintaining protein stability, ligand/protein interactions and provide passage to ions across membranes [5].

Several algorithms and in silico methods have been developed specifically to investigate protein cavities and their associated chemical spaces. These algorithms can be broadly classified into three classes based on their hypotheses, namely evolution-based, energy-based, and geometry-based. Evolution-based algorithms such as genetic active site search (GASS) [6], catalytic site identification (CatSId) [7], conservation surface mapping (ConSurf) [8], and amino acid pattern search for substructure and motifs (ASSAM) [9] rely on the sequence and structure similarity of conserved residues. Their performance depends on the availability of homologous proteins and the quality of the alignment method [4, 10]. Energy-based algorithms (QSiteFinder [11], Grid [12], and AutoLigand [13]) evaluate interaction energies between a probe (of different chemical nature) and protein atoms. Later, protein atoms with optimal interaction energy are clustered to identify cavities. Energy-based algorithms are limited by force field parameterization, scoring function, filtering procedure, and cutoff values. Geometrical-based algorithms analyze the molecular surface of a protein by placing it on a grid or rolling spheres or through geometrical tessellation to locate cavities [4]. Recently, machine learning (ML) and deep learning (DL) algorithms have been employed for predicting protein–ligand binding sites [14].

Different types of geometrical algorithms, such as grid-based algorithms (Cavity Search [15], POCKET [16], LIGSITE [17], CavityPlus [18], etc.), sphere-based algorithms (SURFNET [19], HOLE [20], etc.), surface-based algorithms (CHUNNEL [21], MSPocket [22]) and tessellation-based algorithms (computed atlas of surface topography of proteins (CASTp) [23], Fpocket [24], etc.) have been utilized to identify the cavity. Grid-based methods are sensitive to grid spacing and protein orientation. PyKVFinder is a newly developed grid-based, integrable Python package designed for efficient biomolecular cavity detection [25]. Sphere- and surface-based algorithms do not appropriately define the cavity ceiling and suffer from mouth-opening ambiguity. “Mouth opening ambiguity" refers to uncertainty in predicting the entrance of a protein cavity. Therefore, hybrid algorithms (pocket-cavity search application (POCASA) [26], McVol [27], CAVER [28], etc.) have been developed by utilizing a combination of grid and sphere/surface methods. The geometrical hybrid algorithms resolve the limitations of the individual approach, but the combination increases the complexity and lengthens processing time.

The concept of tessellation was introduced by Edelsbrunner and his coworkers to identify protein cavities [29]. Different geometrical tessellation-based methods, such as Voronoi diagrams, Delaunay triangulation, α shapes, β shapes, and Apollonius graphs, have been utilized for cavity determination [29]. Delaunay and Voronoi diagram-based methods utilize a set of points to detect protein cavities. The α-shape method, *i.e.,* Automated PROtein POcket Search (APROPOS) [30], uses the alpha scan to remove Delaunay triangulation having a circumcircle greater than an α value. However, it is possible that triangulation may vanish from the cavity as a result of unsupervised removal [4]. CAVE [31] methods use Delaunay triangulation to identify voids in proteins. MOLE uses Voronoi tessellations to predict channels, tunnels, and voids [32]. CAST utilizes a combination of a Voronoi diagram and Delaunay triangulation to obtain a dual subcomplex of protein. It implements discrete flow on the complementary dual subcomplex, which states that the obtuse triangle flows to its neighboring triangle, whereas the acute triangle acts as a sink for the obtuse flow. The discrete flow is limited by the absence of an acute triangle in a cavity and leads the obtuse flow to infinity [4, 23, 33]. Fpocket builds a Voronoi diagram of the center of protein atoms, and different radii of alpha spheres are placed at the Voronoi vertex to identify the cavity. The alpha spheres are added at each Voronoi vertex, regardless of their occurrence inside the cavity, thereby increasing the computational cost [24]. The above mentioned methods are points-based, which utilize a uniform van der Waals radius for all atoms. The other tessellation methods, such as Medak et al. [34] and Kim et al. [35], were developed that use weighted Voronoi diagrams or weighted Delaunay triangulation methods for cavity identification. The weighted geometrical methods consider the distinct van der Waals radius for each atom instead of the center of the atom. Furthermore, Apollonius diagrams and graphs were also used by some tessellation algorithms, such as VoroProt [36] and Lindow et al. [37]. Generally, the weighted geometrical algorithms are more complex than the point-based geometrical methods.

This work introduces a new cavity prediction algorithm, known as the “Flow Transfer Algorithm”. Delaunay triangulation is set of tetrahedra formed from a set S of points in R^3^ (real number) such that their circumspheres (the spheres passing through all four vertices of a tetrahedron) do not contain any point of S in their interior [38]. Delaunay triangulation partitions the convex hull of set S into a regular mesh of polytopes. First, the algorithm identifies delimiter tetrahedra by peeling off the convex hull and distinguishes susceptible tetrahedra (i.e., more prone to be situated within cavities) among the regular mesh of tetrahedra. Furthermore, each susceptible tetrahedron is accurately scanned using the maximum circle radius (MCR) to cluster all other tetrahedra present inside the cavity. The MCR signifies the maximum 2D radius of the circle that can pass through a face after considering distinct circles at its vertices.

Generally, the 2D weighted Delaunay triangulation is defined for a set C of circles is denoted by C_i_ = (c_i_, r_i_), where the center c ∈ R^2^, radius r ≥ 0 and i ∈ I (integer number), such that the orthogonal circle passing through the vertex circles of the triangle does not contain any circle from set C. Its 3D implementation will be more computationally expensive, as spheres are used rather than circles [38]. Therefore, instead of applying 3D weighted Delaunay triangulation for all protein atoms, the algorithm utilizes the 2D MCR criteria at the faces of tetrahedron to transfer seed flow (originated from a susceptible tetrahedron) to its neighboring tetrahedra. The MCR has four primary advantages: (a) considers different van der Waals radii for protein atoms, (b) reduces the dimension complexity as each face of tetrahedron is projected on the 2D plane to calculate the MCR, (c) performs multidirectional cavity scan, and (d) the MCR is equivalent to the probe radius used by other algorithms.

The proposed algorithm can identify different types of cavities (such as voids, clefts, pockets, and channels), considering van der Waals radii, and quickly processing large proteins. The algorithm can also consider hydrogens, cofactors, and bound ligands, along with the protein atoms based on user specifications, to identify more accurate and reliable cavities. The upcoming sections detail the working principle of the proposed flow transfer algorithm (FTA), the web architecture of Cavity Recognition Assisted by Flow Transfer algorithm (CRAFT), testing CRAFT with various proteins to identify different cavities, time complexity assessment for each step, and a run-time comparison with Fpocket [24] and CavVis [5] tools.

## 2D aspect of the algorithm

The proposed algorithm implements Delaunay triangulation because of its unique feature of partitioning convex hulls into regular simplexes. Regular simplexes, such as triangles and tetrahedra, have a fixed number of faces, edges, and vertices. Delaunay triangulation is the dual graph of the Voronoi diagrams. However, the Voronoi diagram partitions a set of points into a mesh of polygon Voronoi regions such that the edges of a Voronoi region are given by its neighbors [38]. The proposed algorithm utilizes the Delaunay triangulation instead of its dual Voronoi diagram because regular simplexes are simpler to manage than the Voronoi regions, and due to no overlap between the simplexes, transition from one simplex to another is easier. Hence, a basic idea was initially developed to estimate the empty space between a set of points using the 2D Delaunay triangulation.

**Fig. 1:**
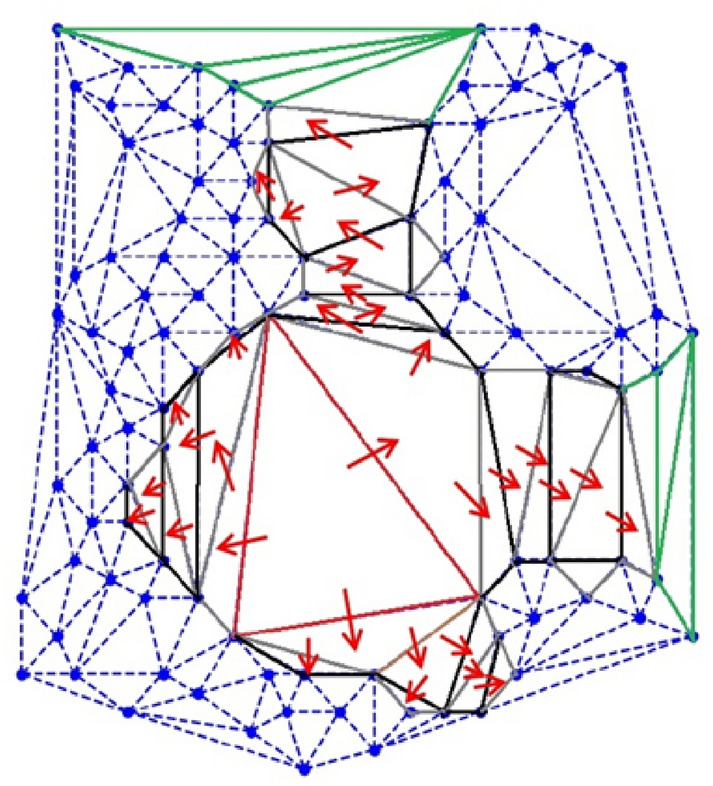
Delaunay triangulation of the considered points was created using the Python scipy (geometrical) package. Each point has an invisible circle that represents the protein atom. The green triangle represents the surface triangle. The red arrow represents the flow from the red seed triangle to the entire cavity/empty space through edge-sharing triangles represented in alternate colors (grey and black)

Consider a point set S in R^2^, such that each point has an invisible circle of different radius to represent the protein’s spherical atoms. The close proximity of points in the set S represents the bonded atoms of the protein. Delaunay triangulations are constructed for set S such that their circumcircle does not contain any point of set S in their interior. An unstructured mesh of Delaunay triangles comprises of small and large triangles (Fig. 1). Typically, small area triangles are due to bonded atoms (proximity points), and large area triangles are used as starting points to estimate the empty space, *i.e.,* the cavity present inside set S, which further comprises large and small triangles (grey and black color). Suppose that the cavity scan began from the largest red triangle (Fig. 1) to cluster other triangles in empty space, the red triangle is examined along its three edges. If the edge is large enough in length, after subtracting the respective circle radii, then flow transfers to the neighboring triangle. Likewise, the flow continues until it fails the edge criteria or meets the triangles of the convex hull, *i.e.,* the surface triangle. The same approach is utilized to estimate the protein’s cavities using the 3D Delaunay triangulation.

## Flow transfer algorithm

3D Delaunay triangulations (point-based) of a set S of atom coordinates of protein in R^3^ have been constructed such that its circumspheres do not contain any point of S in their interior. It gives an unstructured mesh of tetrahedra (four vertex atoms, six edges, and four faces) simplexes. The algorithm begins with identifying delimiter and susceptible tetrahedra.

### Delimiter tetrahedra

Delimiters are used to eliminate the mouth-opening ambiguity of the cavity, which arises due to several depressions present on the protein surface. A convex hull is used to define the delimiters for the points of set S. A tetrahedron with fewer than four neighbors constitutes the convex hull of set S. Convex hulls alone cannot provide sufficient delimiters due to protein surface depression; therefore, identified convex hull tetrahedra are further peeled off to extract new tetrahedra. The peeling process is continued until the newly extracted tetrahedra has circumsphere radii greater than the user-defined peeling-off radius (default 10 Å). The collection of convex hull and extracted tetrahedra is referred to as surface tetrahedra. Tetrahedra originating from the vertex atoms of surface tetrahedra act as delimiter tetrahedra that help terminate the seed flow. The size of the cavity can increase or decrease depending on the user-defined peeling-off radius.

### Susceptible tetrahedra

Susceptible tetrahedra are tetrahedra with a high propensity to be present inside a cavity except the delimiter tetrahedra. Small volume and circumsphere radius tetrahedron simplexes are present between the bonded atoms, whereas larger tetrahedra simplexes are observed between the unbonded atoms (atoms at a distance), which are likely to be inside the cavities. The tetrahedron volume and circumsphere radius (default 4 Å) are used to identify susceptible tetrahedra. Susceptible tetrahedra supplies seed for the cavity scan, as it pinpoints all prior locations inside the protein where a cavity might be present.

After the identification of delimiter and susceptible tetrahedra from the mesh of 3D Delaunay triangulations of set S, a flow transfer algorithm-based cavity scan is initiated in three steps:

- a) Initiation

**Fig. 2:**
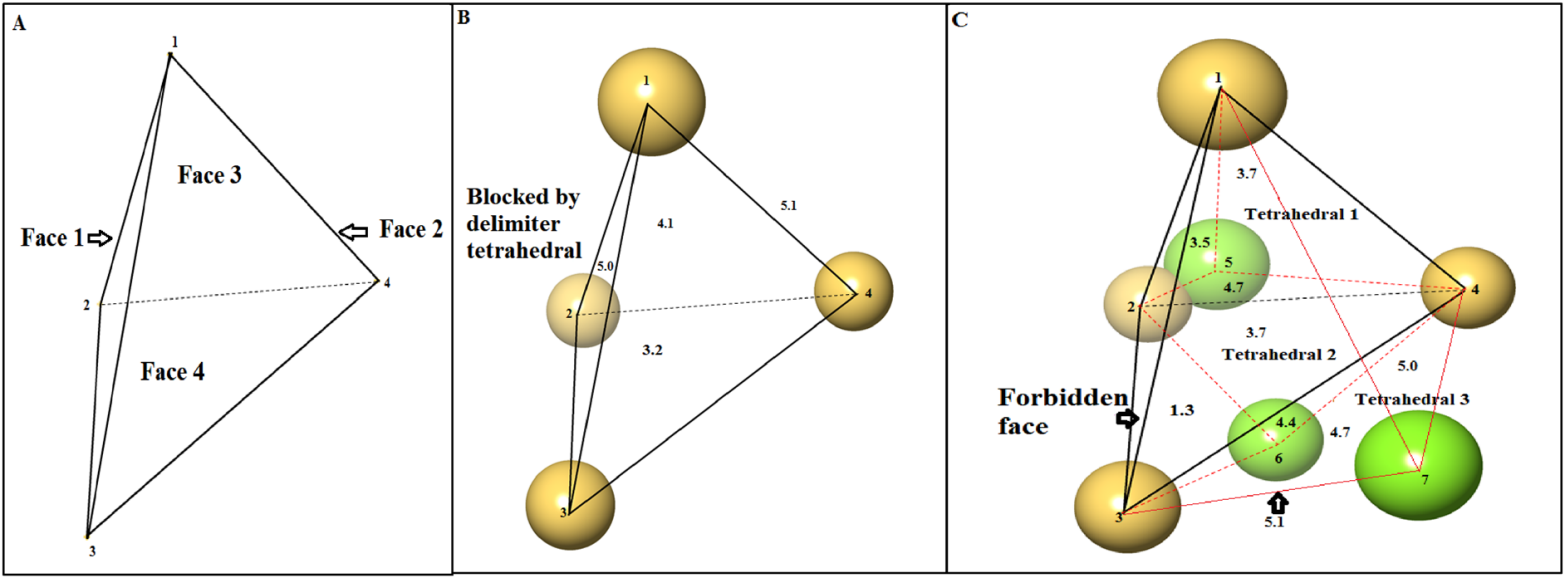
A diagrammatic representation of flow propagation from the seed tetrahedron to its neighboring tetrahedra through its faces. The values are given in angstroms (Å) and represent the maximum radius of the circle (MCR) that can pass through a face after considering the van der Waals radii of atoms at their vertices. If a user-defined MCR (1.4 Å) is used to scan a cavity, the algorithm **A** Selects a seed tetrahedron from the susceptible tetrahedra inside the cavity. **B** Flow transfer to three neighboring tetrahedra (T1, T2, and T3) from faces (Face 3, Face 4, and Face 2) of the seed tetrahedron, respectively. The flow is transferred across all four faces of the seed tetrahedron, as the default MCR is greater than the user-defined MCR. However, the neighboring tetrahedron of Face1 is found to be a delimiter tetrahedra; therefore, flow cannot be transferred. **C** Flow transfer through the eight faces of tetrahedra T1, T2, and T3, whereas one face of tetrahedron T2 is forbidden because the MCR is less than the user-defined radius

The algorithm starts the cavity scan from a seed tetrahedron supplied by the susceptible tetrahedra using a user-defined MCR (default 1.4 Å), which is equivalent to the probe sphere radius (Fig. 2A), and transfers the seed flow to the neighboring tetrahedra. The algorithm represents the probe sphere in two dimensions, *i.e.,* the largest radius of a circle of the probe sphere, *i.e.,* MCR.

- b) Propagation

Seed tetrahedron has a high propensity to be present inside a cavity. Therefore, to cluster other tetrahedra of the cavity, the seed tetrahedron is scanned across four faces. The MCR is calculated for each face of the seed tetrahedron. To transfer the seed flow to its corresponding neighbor, a face must meet the prerequisite listed in Eq. (1).

1 $$calculated\, Maximum\, Circle\, radius\,>\,user\, defined\, Maximum\, Circle\, Radius$$

If a face satisfies the MCR condition, then the face is allowed to transfer seed flow; otherwise, the face is forbidden for transfer. The seed tetrahedron can transfer its flow to four faces sharing neighbor tetrahedra, while all other tetrahedra originating from the seed tetrahedron can travel into its three-face sharing tetrahedra, as the fourth neighbor would be the one from which it originated. The seed flow continuously transfers to new face-sharing neighboring tetrahedra and generates a cluster of all tetrahedra present inside a cavity (Fig. 2B, C). The seed flow transfer process continues until the face is not forbidden.

- c) Termination

The seed flow transfer is terminated either by a forbidden face (*i.e.,* the calculated MCR is less than or equal to the user-defined MCR) or by the presence of delimiter tetrahedra (Fig. 2B, C). The seed flow of voids is terminated by forbidden faces, as they are surrounded by atoms. Screening for a specific seed tetrahedron or cavity is completed once the seed flow has entirely stopped. A cluster of all tetrahedra originating from the seed tetrahedron represents a cavity. The cluster may also contain susceptible tetrahedra other than the seed tetrahedron; therefore, to avoid rescanning the same cavity, such tetrahedra are removed from the susceptible tetrahedra.

After the termination of a seed flow, the susceptible tetrahedra supply a new seed tetrahedron to find other cavities inside the protein. Likewise, the protein cavity scan continues until all susceptible tetrahedra are scanned. An intricate working of the designed algorithm is thoroughly delineated in the pseudocode, provided in Sect 2 of the Additional file 1.

## Maximum circle radius (MCR)

As defined above, MCR is the largest possible circle that can pass through the face of a tetrahedron after considering different radii of atoms at its vertices. Tetrahedron faces are projected onto a 2D plane to estimate the MCR that can fit within an arrangement of three atoms of different radii (*i.e.,* vertex atoms of a face). It enables multidirectional scanning of the seed flow, and its directions depend on the newly extracted faces of tetrahedra. As discussed, the seed flow is transmitted to neighboring tetrahedra through the faces if the faces meet the MCR criteria; therefore, it is essential to estimate the MCR of a face.

**Fig. 3:**
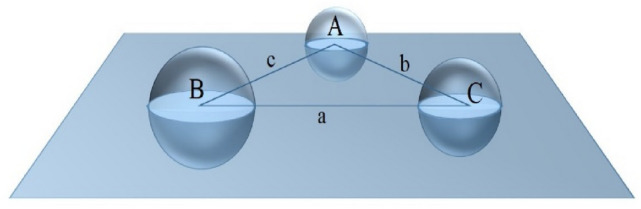
A diagrammatical illustration of the face of a tetrahedron having three atoms of different radii at its vertices in the XY plane. The maximum radius of the sphere that can pass through them is equivalent to MCR that can pass through △ABC. The a, b, and c are the Euclidean distance between the centers of the spheres A, B, and C, respectively

Consider a triangle △ABC in the XY plane represents a tetrahedron face, such that its vertices represent atoms (*i.e.,* spheres) with different van der Waals radii (Fig. 3). Face atoms can be present in different arrangements inside the triangle, such as acute, right, and obtuse angles. Each vertex of the △ABC has two protruded hemispheres, one above and one below the plane. The MCR that can pass through the arrangement of atoms of △ABC can be easily estimated by cutting off the hemispheres of the vertexes. The three-dimensional face of the tetrahedron is transformed into a triangle with circles at its vertices. Now, circles (shown in light blue color in Fig. 3) are present at the vertices of the triangle, and the MCR that passes through △ABC will represent the radius of the maximum sphere. Therefore, each tetrahedron face is projected on the XY plane to transition from a tetrahedron to its neighboring tetrahedron.

Consider a face having three atoms A, B, C ∈ R^3^ at vertices with radii r_1_, r_2_, and r_3_. The Euclidean distance of edges is used to project face vertices on the XY plane.

**Fig. 4:**
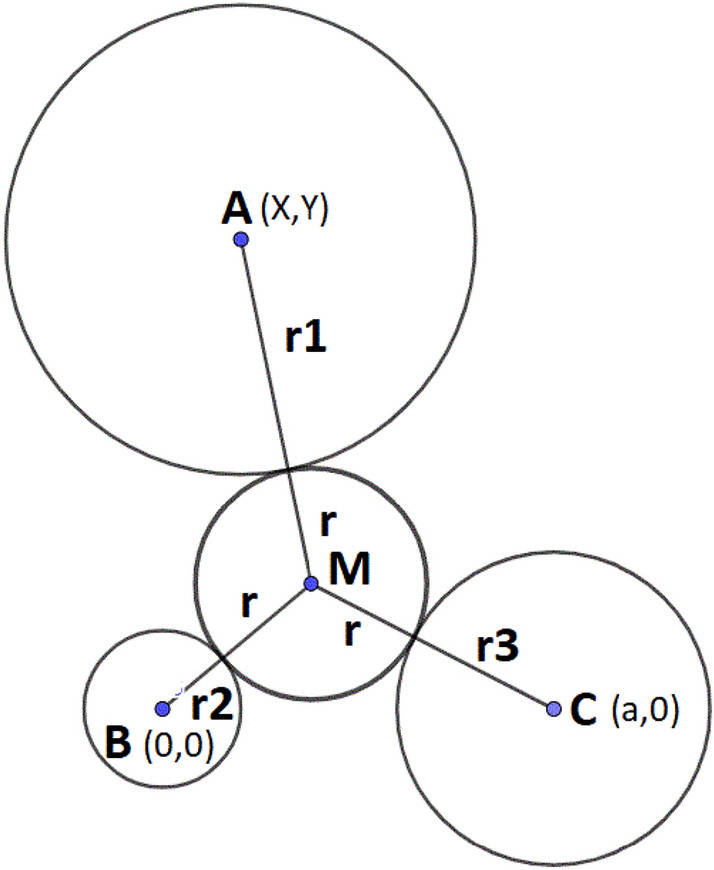
2D projection of a face of tetrahedron on the xy plane

Assume a vertex B at origin (0,0), vertex C at x-axis (a, 0), and third vertex A (X, Y) can be calculated from, *i.e.,* the Euclidean distance of edges AC and AB, respectively, of the considered face (Fig. 4).

In Fig. 4, M (x,y) represents the Maximum Circle Radius (MCR), the radius of a tangent circle that can pass through the vertices A, B, and C. The radius of MCR (r) can be easily determined by solving the subsequent set of Euclidean distance equations (detailed formulas are given in Sect 1 of the Additional file 1).

2 $${x}^{2}+{y}^{2}={({r}_{2}+r)}^{2}$$

3 $${(x-a)}^{2}+{y}^{2}={({r}_{3}+r)}^{2}$$

4 $${(x-X)}^{2}+{(x-Y)}^{2}={({r}_{1}+r)}^{2}$$

The equations mentioned above offer a solution for the Maximum Circle Radius (MCR), denoted as 'r' in various arrangements of protein’s atoms, including acute, right, and obtuse configurations. However, they fail when all three vertex circles (atoms) overlap, resulting in no available space to accommodate MCR. It is likely a situation that can occur inside the protein’s atom coordinates. To address this, a pre-check is performed at the face to detect all-atom overlap, and in that case, the MCR is set to zero.

## CRAFT architecture

CRAFT is a client–server web application that implements the Python Flask application on the server-side code and HTML, CSS, and JavaScript on the client-side code. The HTML is used to design the webpage layout, CSS style for visual enhancement, and JavaScript for client-side interactions, form validation, and asynchronous JavaScript and XML (AJAX) requests. Python Flask implements several routes and corresponding handlers to process incoming requests and render HTML files. AJAX makes asynchronous requests between the client and server without reloading the entire page. JavaScript/jQuery handles AJAX requests, event handling, and dynamic webpage updates. The 3Dmol.js library is used to render the 3D structure of the protein and cavities on the webpage. Client and server communication occurs over the HTTP protocol, with AJAX requests sending data from the client to the server and receiving responses asynchronously. AJAX and jQuery support modules for chain, ligand, and alternate position selection. User inputs are validated on the server side, and the server code is optimized for efficient processing and response time.

CRAFT's client-side interface includes navigation buttons for Home, Computation, Alerts, Features, and Concept. The computation panel accepts a standard .pdb file as input and default parameters such as MCR (default 1.4 Å), peeling off radius (default 10 Å), susceptible radius and volume (default 4 Å), false extended boundary, false extended forbidden face, and number of cavity atoms (default 15). The default “Extended Boundary” setting removes larger and smaller tetrahedra from the convex hull, but the changes from user end in settings removes larger tetrahedra only. The default “Extended Forbidden Face” setting terminates the algorithm when encountering a forbidden face and includes a shared tetrahedron with the forbidden face when modified. Users can adjust these parameters based on their prior knowledge. CRAFT initiates cavity scanning based on user inputs. Various properties of the predicted cavities are tabulated and given in descending order of volume. Users can select a cavity to view the corresponding 3D visualization, percentage contribution graph, highlighted residues on the sequence, and cavity details (residue number, chain, atom type, atoms). Alerts have been implemented to inform users about input and output-related warnings.

## Command line python program

A command line python program has also been developed, which is publicly available at https://github.com/PGlab-NIPER/CRAFT, with a user manual. The program employs the “Flow Transfer Algorithm” to identify and characterize protein cavities. The user manual provided with installation instructions cover Python, SciPy, and FreeSASA, ensuring a smooth setup process. Users are guided through downloading, unzipping the CRAFT tool files, and saving PDB files in the designated folder. Running the algorithm via the command prompt is straightforward, as users enter the command “Python main.py” and follow on-screen prompts. The command line tool can process both single and multiple .pdb files, storing the results in dedicated folders. The tool subsequently provides detailed cavity information, including volume, residue details, atom properties, and physicochemical characteristics, in descending order of cavity volume.

## Features

### Multidirectional cavity scan

The cavity scan starts from the seed tetrahedron, supplied by the susceptible tetrahedra. To obtain an idea about the directions of cavity scanning, termination criteria (*i.e.,* encounter with either forbidden face or delimiter tetrahedra) were omitted. So that the seed flow can spread out in four directions, away from the core of the seed tetrahedron's faces. Furthermore, each direction is split into three new directions by the faces of the neighboring tetrahedra of the seed. This is because the seed tetrahedron can transfer its flow to four-face sharing tetrahedra; however, all other tetrahedra that have descended from it can only move into their three-face-sharing tetrahedra, as the fourth neighbor would be the one from which it descended.

Likewise, cavity scan directions can be observed as 1, 4, 12, 36.., 3(n-1), and the direction is indicated away from the core of the tetrahedron's face. After the seed tetrahedron transitions into four directions, all subsequent tetrahedra will follow the geometrical progression (GP) series to transit the seed flow into neighboring tetrahedra with a GP ratio of 3. The scanning follows the geometrical progression until it encounters a forbidden face or delimiter tetrahedra. It is clear from the discussion that the algorithm performs multidirectional cavity scanning. FTA does not need a defined vector, as a face can only be shared by two tetrahedra, *i.e.,* a property of Delaunay triangulation. However, the scanning directions depend on the cavity size and the termination criteria. This feature makes the algorithm fast and efficient in predicting protein cavities.

### CRAFT (cavity recognition assisted by flow transfer algorithm)

CRAFT, a user-friendly web interface, has been developed for the Flow Transfer algorithm, which makes this algorithm easily accessible to computational biologists and pharmaceutical scientists. It can be used to predict surface atoms of distinct cavities, such as voids, clefts, pockets, and channels. It facilitates the user in choosing non-hydrogen, hydrogen, cofactor, and ligand atoms to accurately predict protein cavities. It can handle different van der Waals radii of atoms using the MCR concept. It also provides a 3D panel to visualize the identified cavities of proteins, and the abovementioned cavity properties. It generates a percentage contribution graph for each identified cavity and highlights associated residues on the protein sequence. A unique combination of Delaunay triangulation and the MCR for a given protein ensures the accuracy and reliability of the algorithm.

## Cavity properties

Protein cavity features (such as hydrophobicity, hydrogen bonding, buriedness, cavity size, and enclosure) play an important role in the molecular recognition of ligands and predicting functional sites. Several studies have assessed the functional binding site present inside a protein [39]. Generally, the largest and deepest cavity acts as the functional site [40]. The cavity properties can be utilized for cavity-based classification of proteins, to understand conformation changes and the effect of allosteric sites on functional sites, and to design novel drug-like molecules. The cavity properties calculated by the CRAFT are described below:

- a) Cavity structure

It contains information about atoms, residues, residue numbers, chains, and atom types inside a cavity. The cavity residues are also highlighted on the protein sequence.

- b) Solvent Accessible Surface Area (SASA)

FreeSASA [41] is an open-source C-library and Python module used to calculate the SASA of all cavity atoms using a probe equivalent to the MCR. The total SASA of a cavity is given by Eq. 5.

5 $${T}_{SASA}=\sum_{i=0}^{n}{SASA}_{i}$$

where n is the total number of cavity atoms, and SASA_i_ is the SASA of an individual atom.

Similarly, the polar/nonpolar SASA is calculated by summation of the SASA of polar/nonpolar atoms inside the cavity.

- c) Donor and Acceptor

The donor/acceptor atoms are electronegative atoms inside the cavity. The number of hydrogen (nH) atoms attached to an electronegative atom can be calculated by Eq. 6.

6 $$number\, of\, Hydrogen\, (nH)=Valency-Bond+Charge$$

Three conditions are used to classify an atom as a donor/acceptor:

1. If nH is equal to zero, then the atom is the acceptor.
2. If nH is nonzero and the charge on the atom is positive, then the atom is a donor.
3. If nH is nonzero and the charge on the atom is nonpositive, then the atom is both donor and acceptor.

- d) Aromaticity Cavity atoms are scanned to find aromatic rings from phenylalanine, tryptophan, tyrosine, and histidine. Histidine is considered aromatic with a net charge ≤ 0 (*i.e.,* basic pH).

- e) Cavity volume

Cavity volume is defined as the total volume of all tetrahedra minus the volume of the portion spheres present at the tetrahedra vertices. It will give an idea about the empty space available inside the cavity.

- f) Percentage contribution per residue

It is defined as the percentage of the number of residue atoms present inside a cavity to the total number of residue atoms. It represents the percentage involvement of each residue present inside the cavity.

- g) Kyte-Doolottle (kd) score

The Kyte-Doolittle scale comprises of hydrophilic and hydrophobic properties of the side chain of 20 amino acids and represents the average hydrophobicity of each amino acid. The scale showed good correlation between the estimated value and known experimental structures for soluble and globular proteins [42]. The Kyto-Doolittle cavity score is defined as the summation of the Kyto-Doolittle score of each cavity residue multiplied by its percentage contribution to the cavity (Eq. 7). The positive values on the kd scale signify the hydrophobic nature of the residue and vice versa.

7 $$kd\, cavity\, score\,=\,{\sum }_{n=1}^{m}\frac{\mathrm{kd\, of\, n\, residue }* percentage\, contribution\, of\, n\, residue}{100}$$

- h) Exposure and enclosure

Exposure is defined as the ratio of the number of atoms present at the cavity entrance to the total number of cavity atoms. The entrance atoms are determined by identifying unshared faces of cavity tetrahedra that are not forbidden faces. The enclosure is defined as the ratio of non-entrance atoms to the total number of cavity atoms.

- i) Number of cavity mouths

To determine the number of cavity mouths, unshared faces of cavity tetrahedra that are not forbidden are grouped based on their continuous edge sharing. The total number of such groups represents the cavity mouth. A smaller MCR shows more cavity mouths, while a larger MCR shows fewer mouths for a cavity.

The CRAFT ranks cavities present inside a protein based on their volume. This signifies that a cavity with a larger volume would rank higher and vice versa.

## Results and discussion

CRAFT was validated for accuracy on different crystal structures consisting of diverse forms of protein, such as monomer, homomeric, and heteromeric forms. CRAFT was utilized to search for different types of cavities, such as voids, pockets, channels, and surface pockets. A cavity search was also performed in the presence of cofactor and hydrogen atoms. The algorithm was executed with default parameters (MCR = 1.4 Å, peeling off radius = 10 Å, susceptible volume and radius = 4 Å, extended boundary = false, extended forbidden face = false, and cavity atoms = 15) unless specified.

- a) Identification of the functional pocket and surface cavity in monomers

**Fig. 5:**
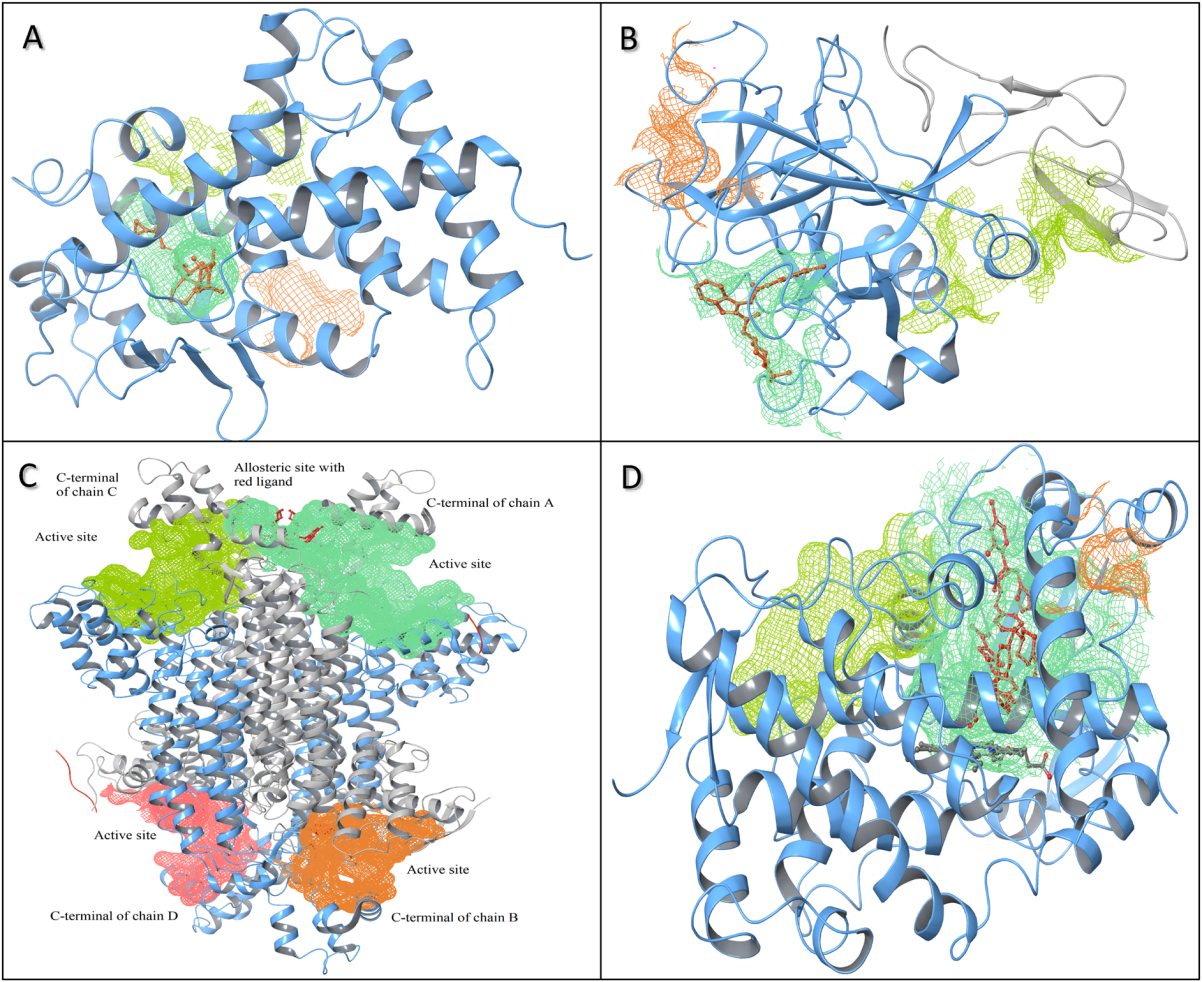
The top three cavities are represented in aquamarine, yellow-green and orange color based on their volume in descending order. **A** vitamin D nuclear receptor (PDB ID: 1S19), **B** factor Xa (PDB ID: 2BQW), **C** fumarate hydratase (PDB ID: 5F91), **D** CYP3A4 (PDB ID: 2V0M, A chain). Ligands and cofactors are represented in red and gray, respectively, inside the functional cavity

CRAFT was tested using two monomer protein structures of vitamin D nuclear receptor in complex with calcipotriol (PDB: 1S19) and 2-carboxyindole-based factor Xa inhibitors (PDB: 2BQW)) belonging to the gene regulation and hydrolase class of *Homo sapiens* [43, 44]. The CRAFT ranked the detected functional cavities as first, represented by the aquamarine shading (Fig. 5A&B). A longitudinal empty space around the predicted functional cavity in the vitamin D nuclear receptor suggests that further chain elongation is possible in molecules such as calcipotriol (Fig. 5A). The cavity shape also suggests that linear molecules can easily access the cavity compared to branched molecules.

In another case study, a highly solvent-exposed functional site of factor Xa poses a challenge for cavity prediction methods such as DoGSite and CAVIAR, which were unable to detect it [45]. CRAFT also predicts an incomplete cavity with default parameters for the crystallized ligand hanging outside the cavity. However, it identifies the complete portion of the functional site with the “Extended boundary” option, despite the high solvent exposure (Fig. 5B). Therefore, CRAFT enables flexibility to adjust various parameters of FTA to accurately predict cavities in diverse proteins, distinguishing it from other available tools.

- b) Identification of allosteric site and cavity involving cofactor as an integral component

CRAFT was tested using fumarate hydratase (PDB ID: 5F91, open conformation having ligand at allosteric site) from *M. Tuberculosis,* which is a homotetramer reported to have four active sites and two allosteric sites [46]. Its allosteric sites show selective competitive inhibition toward *M. tuberculosis,* as allosteric ligand binding induces conformation changes such that the enzyme remains in the open conformation and prevents simultaneous ligand binding to the active site [46]. CRAFT was able to identify four well-defined cavities (Fig. 5C). It includes two larger cavities (aquamarine and green‒yellow) at the C-terminal end of the A and C chains, in contrast to two smaller cavities (orange and pink) at the C-terminal end of the B and D chains (Fig. 5C). This disparity in volume may be attributed to ligand (red) binding at the allosteric site, inducing conformational changes in loop regions of the C-terminal domain and enlarging the cavity size. Additionally, these conformational changes prevent ligand binding at the active site. The results indicate that CRAFT is a valuable tool for identifying allosteric sites and elucidating protein conformational changes associated with cavities.

To determine CRAFT’s utility in identifying cavities in proteins associated with cofactors, the human oxidoreductase enzyme CYP450 3A4 (PDB: 2V0M, chain A), characterized by the presence of a heme catalytic center [47], was used. The CRAFT has successfully ranked the functional site (aquamarine) as first, taking into account the attached cofactor, while other tools, such as CASTp, were unable to do so, resulting in extra cavity atoms due to the absence of cofactor. The functional site of the CYP3A4 enzyme was found to have two ketoconazole molecules (yellow and green) bound in an antiparallel orientation (Fig. 5D). Therefore, CRAFT enables accurate identification of protein cavities even in the presence of cofactors.

- c) Identification of biologically significant cavities and crucial residues in the presence of hydrogen atoms

**Fig. 6:**
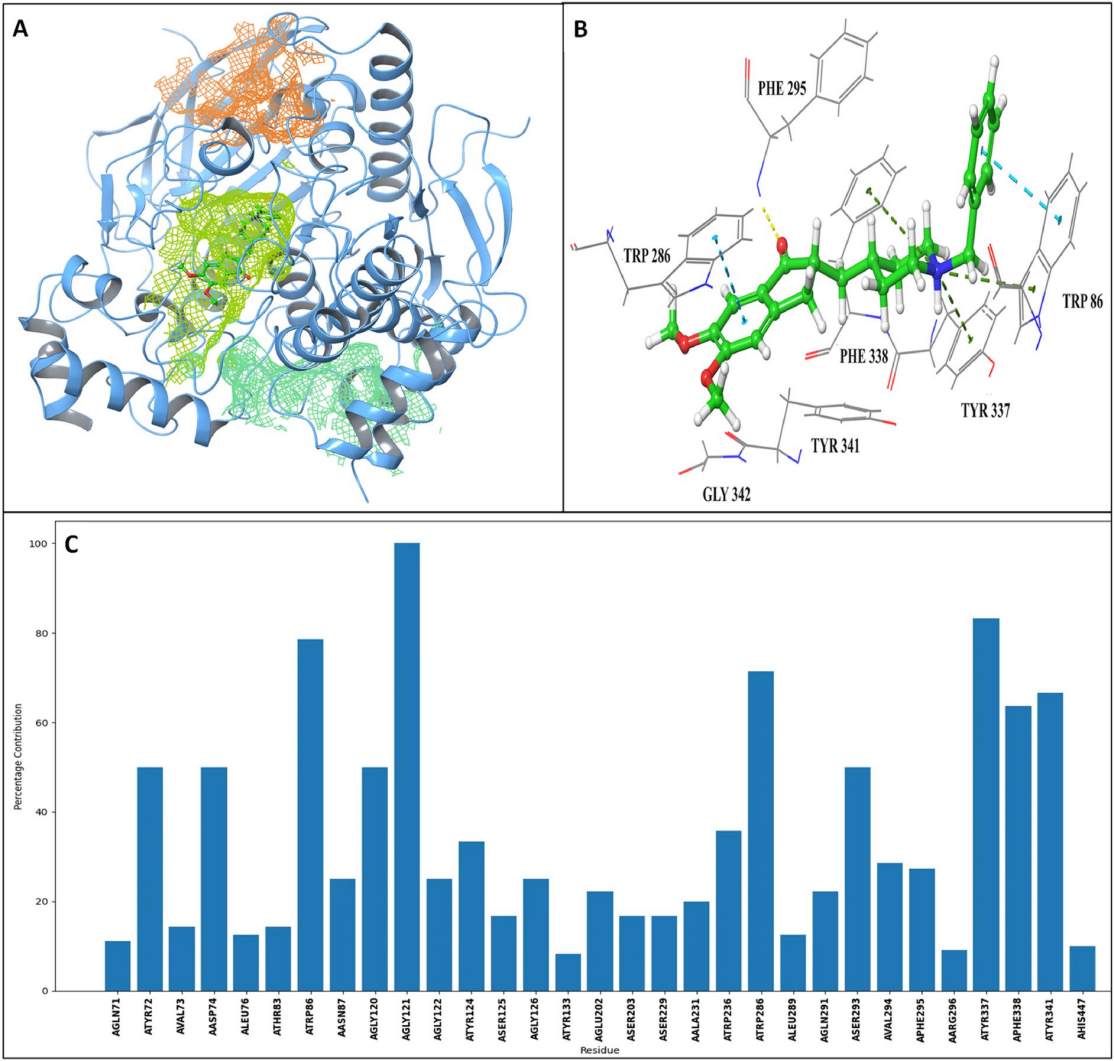
**A** The top three cavities identified by CRAFT are represented for acetylcholine esterase (4EY7 (A)) in aquamarine, yellow‒green and orange color based on their volume. **B** Docking interaction of donepezil inside the functional site, **C** Percentage contribution graph for the functional site

The CRAFT was implemented to analyze cavities inside the acetylcholine esterase (PDB ID: 4EY7, chain A), a hydrolase enzyme present in *Homo sapiens* used as a target for Alzheimer's disease [48]. The cavity scan was performed with the inclusion of hydrogen atoms using the MCR (1 Å) and peeling off radius (8 Å). The functional cavity for the drug donepezil was ranked second by the CRAFT, depicted in yellow-green color (Fig. 6A). Notably, the presence of five aromatic rings inside the second cavity suggests its likelihood of being a functional cavity. Thus, cavity properties can also aid users in predicting the functional cavity, as volume-based ranking is not always reliable.

A percentage contribution graph for the functional site of acetylcholine esterase highlights residues with high cavity occupancy, indicating a greater chance of interaction with upcoming ligands. The graph suggests that residues Asp74, Trp86, Trp286, Phe338, and Tyr341 exhibit more than 50% individual cavity occupancy (Fig. 6C). These residues were also observed to interact with donepezil in the docking results (Fig. 6B). The study emphasizes the significance of hydrogen in cavity prediction, as the absence of hydrogens leads to the prediction of some unnecessary cavity residues. The percentage contribution graph is valuable in comprehending the crucial residues within a cavity.

- d) Identification of channels and pockets in homomeric and heteromeric proteins

**Fig. 7:**
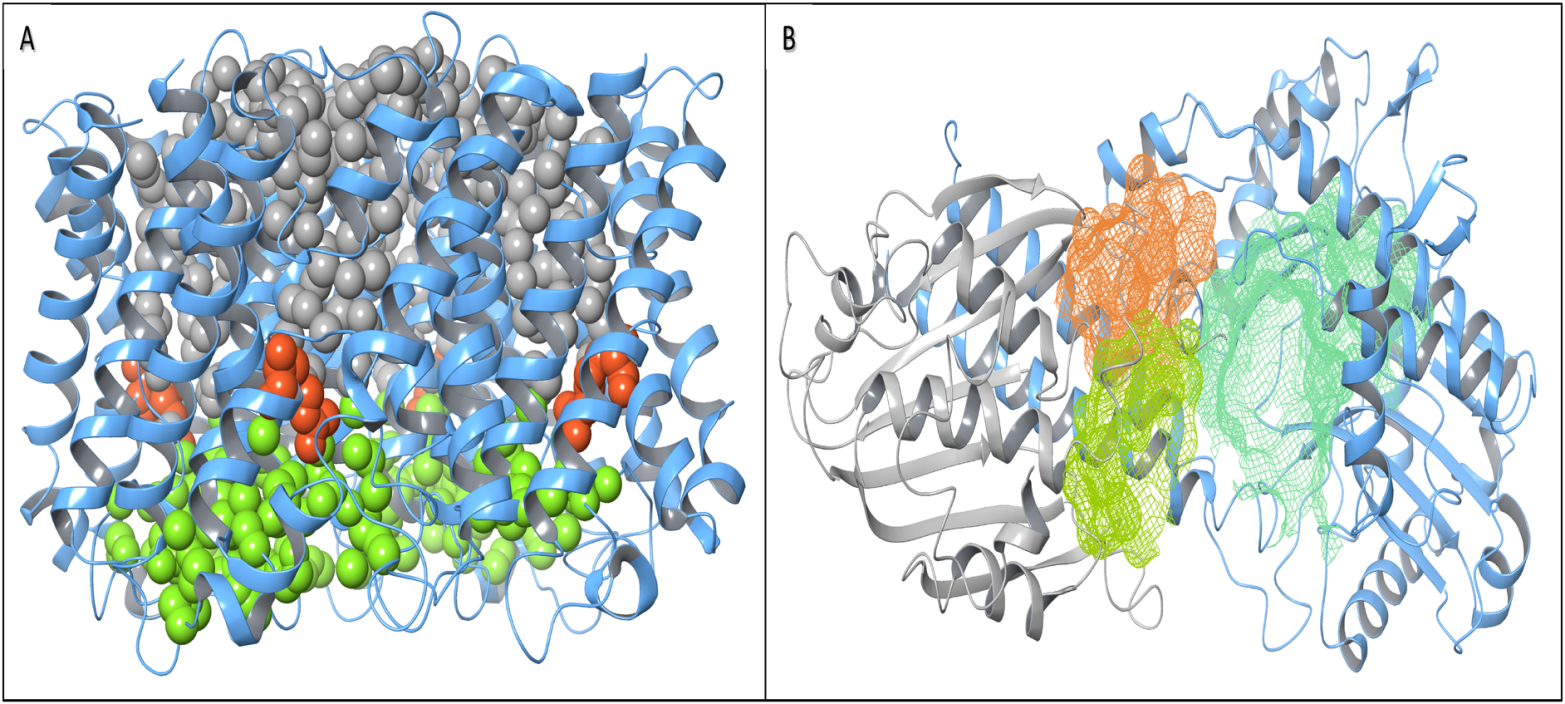
**A** Aquaporin Z (2ABM); each channel is represented in two colors (upper portion in gray and lower portion in green). The Arg189 residue of each channel is shown in red spheres. **B** Top three cavities are represented for the Pnkp/Hen1 heteromer complex (4DRF) in aquamarine, yellow‒green and orange color based on their volume

To identify channels in homomeric proteins, Aquaporin Z (PDB ID: 2ABM), a homotetramer present in the plasma membrane of *Escherichia coli,* known for fundamental osmoregulation, was used. The crystallized aquaporin Z structure is composed of four water-conducting channels, and each channel is constricted at the Arg189 residue. The side chain of the Arg189 residue shows two distinct conformations, but it is not clear whether these conformations are responsible for the opening and closing of channels or only represent its dynamic nature during water permeation [49]. The CRAFT results were found to be in coherence with the reported study, as each channel (among four channels) was observed to be segmented into two parts at the Arg 189 residue, shown as a red sphere (Fig. 7A). This suggests that all four channels are close at the Arg189 position. To understand the opening and closing of aqua pores, molecular dynamics studies should be performed along with the constant tracking of channels.

For heteromeric proteins, the bacterial polynucleotide kinase-phosphatase (Pnkp)/hua enhancer 1 (Hen1) complex (PDB ID: 4DRF) known to perform RNA ligation in *Acetivibrio thermocellus*, an anaerobic bacterium, was used to identify the cavity [50]. The CRAFT identified three cavities (Fig. 7B). The functional cavity was not confirmed, as RNA was absent in the complex, but the largest aquamarine color pocket most likely to bind to the RNA molecule ((Fig. 7B).

## Time performance

To evaluate the performance of the proposed algorithm in terms of the time it takes to predict the cavity, testing was performed on an HP ProDesk 600 equipped with a 2.90 GHz Intel core i7 processor, 64 GB RAM, and an Intel UHD Graphics 630 running the Windows 11 Pro operating system. A single-thread program of the flow transfer algorithm (FTA) was run on a central processing unit (CPU) using the command prompt. The time complexity for the case studies is given in Table 1. The total time depends on the number of atoms present in a protein. It was observed that steps such as Delaunay triangulation, delimiter tetrahedra, and susceptible tetrahedra were relatively quick, while cavity property calculation followed by cavity scan was the most time-consuming.

**Table 1:** Statistics of the time taken by CRAFT at each step to identify cavities and cavity properties using different proteins

Protein	No. of atoms	Delaunay triangulation + Delimiter atoms + Susceptible tetrahedra		Cavity scan		Properties		Miscellaneous		Total time (s)	Time per atom (ms)
		time(s)	%	time(s)	%	time(s)	%	time(s)	%		
1S19	2012	0.03	19.1	0.03	21.1	0.08	52.6	0.01	7.2	0.15	0.08
2BQM	2240	0.03	21.1	0.03	20.4	0.07	48.5	0.01	9.9	0.14	0.06
2VOM(A)	3694	0.05	14.3	0.08	23.2	0.19	55.6	0.02	6.8	0.35	0.09
2ABM	6572	0.09	13.2	0.17	25.3	0.36	54.4	0.05	7.2	0.67	0.10
4EY7(A) + H	8148	0.11	14.7	0.17	23.0	0.41	55.4	0.05	6.9	0.73	0.09
2F91	27325	0.21	12.0	0.44	24.6	1.04	58.8	0.08	4.6	1.78	0.06

The time performance of the FTA was also compared with two existing cavity prediction tools, namely, the Fpocket [24] and CavVis [5] tools (Table 2). The proteins used for the comparison were the same ones used in the Simões et al. study [5] for the CavVis tool. The FTA and Fpocket algorithms were tested on the aforementioned configurations, while the time performance of CavVis was taken from the Simões et al. study, which was performed on an Intel Core i5 processor [5]. CavVis performance was compared with FTA without calculating the cavity properties, while Fpocket was compared with FTA with cavity properties incorporated in its predictions. It is evident that the FTA is a faster algorithm than the Fpocket and CavVis tools, as it performs multidirectional cavity scanning.

**Table 2:** Time performance comparison of the flow transfer algorithm (FTA), CavVis [5], and Fpocket

Protein	No. of atoms	FTA without properties (s)	CavVis(s)	FTA with properties	Fpocket(s)
1VPN	11134	0.62	3.23	1.44	8.22
2YWB	14709	0.97	4.55	2.77	18.83
3M4D	16404	1.26	5.71	4.29	20.71
1TYF	20062	2.42	6.77	6.88	34.79
1XO6	23781	1.96	7.95	4.63	36.64

## Conclusion

Numerous algorithms are available to predict the cavity inside the protein. Each algorithm offers new solutions to predict protein cavities; however, cons are associated with their approach. APROPOS [30] (α-shape-based method) uses unsupervised removal of triangulation with an α value greater than a limit. Certain prediction tools are only used to anticipate specific types of cavities, such as BetaVoid [51] and CAVE [31], used for void prediction, while MOLE [32] can be used for channel, tunnel, and void prediction. The CAST tool can be used to predict all types of cavities, but it implements a complex dual subcomplex approach, and predictions are constrained by the absence of an acute triangle inside a cavity [33]. Fpocket utilizes the same atomic radius for protein atoms, and alpha spheres are added to each Voronoi vertex, irrespective of their occurrence inside the cavity [24]. The implementation of weighted geometrical and Apollonius diagrams [34–37] has solved proteins for distinct van der Waals radii of atoms, but these methods are computationally expensive.

To solve these issues, a new algorithm called the “Flow Transfer Algorithm” has been developed. It identifies delimiter tetrahedra by peeling off the convex hull of the protein atom and performs preliminary cavity scanning to identify susceptible tetrahedra among the regular tetrahedra mesh. Each susceptible tetrahedra is thoroughly scanned using the MCR to cluster all other tetrahedra inside a cavity. The MCR criterion provides information about the forbidden and non-forbidden faces and helps in transferring seed flow to neighboring tetrahedra. To forecast the protein cavities, the algorithm performs multidirectional scanning while considering various van der Waals radii of atoms. The implementation of the MCR in the “Flow Transfer Algorithm” simplifies the algorithm and reduces dimension complexity as each face is projected onto a 2D plane, all while ensuring accurate and reliable results. The algorithm is simpler and takes significantly less time to process large proteins than Fpocket [24] and CavVis [5].

CRAFT, a user-friendly web interface, has been designed for the FTA. It provides the users with different selection modules (chain, ligand, and alternate position modules) and different cavity scanning options (MCR, peeling off radius, extended boundary, extended forbidden face, susceptible tetrahedra volume, and radius) to accurately predict different types of protein cavities (voids, clefts, pockets, and channels). It can be utilized on proteins with cofactors, hydrogens, and ligands as an integral part and provides 3D visualization of protein structure with cavities, cavity properties, percentage contribution graphs, and highlighted residues on the protein sequence for each identified cavity.

The downside of this algorithm is that it cannot give a precise estimate of the protein porosity, but it can identify all cavities that are primarily involved in biological changes. The maximum circle radius (MCR) concept can be extended to other biological entities, such as DNA and RNA, to identify their cavities.

### Supplementary Information

**Additional file 1.** Maximum Circle Radius (MCR) calculations and Pseudocode.

## Data Availability

The data underlying this article is available in the article and Additional file 1. However, CRAFT can be accessed at http://pitool.niper.ac.in/CRAFT, while the command version is available at https://github.com/PGlab-NIPER/CRAFT/.
