# Supplementary material for: CRAFT: a web-integrated cavity prediction tool based on flow transfer algorithm
Source: J Cheminform. 2024 Jan 30;16:12. doi: 10.1186/s13321-024-00803-6 (PMC10829215; doi:10.1186/s13321-024-00803-6)
Supplement: Supplementary file 1 — Additional file 1. Maximum Circle Radius (MCR) calculations and Pseudocode. [file 13321_2024_803_MOESM1_ESM.docx]

**CRAFT: A Web-Integrated Cavity Prediction Tool Based on Flow Transfer Algorithm**

Anuj Gahlawat, Anjali Singh, Hardeep Sandhu, Prabha Garg*

Anuj Gahlawat

Department of Pharmacoinformatics, National Institute of Pharmaceutical Education and Research (NIPER), Sector 67, S.A.S. Nagar -160062, Punjab (India)

Email address: [anujgahlawat512@gmail.com](mailto:anujgahlawat512@gmail.com)

Anjali Singh

Department of Computer Science, Kurukshetra University, Kurukshetra, Haryana (India)

Email address: [miss93anjalighalawat@gmail.com](mailto:miss93anjalighalawat@gmail.com)

Hardeep Sandhu

Department of Pharmacoinformatics, National Institute of Pharmaceutical Education and Research (NIPER), Sector 67, S.A.S. Nagar -160062, Punjab (India)

Email address: [hardeepsndh@gmail.com](mailto:hardeepsndh@gmail.com)

*Prabha Garg

Department of Pharmacoinformatics, National Institute of Pharmaceutical Education and Research (NIPER), Sector 67 S.A.S. Nagar -160062, Punjab (India)

Email address: [prabhagarg@niper.ac.in](mailto:prabhagarg@niper.ac.in); [gargprabha@yahoo.com](mailto:gargprabha@yahoo.com)

**1. Maximum Circle Radius (MCR)**

Consider a face with three atoms A, B, C ∈ R^3^ at vertices having radii r_1_, r_2_, and r_3_. The Euclidean distance of edges is used to project face vertices on the XY plane.


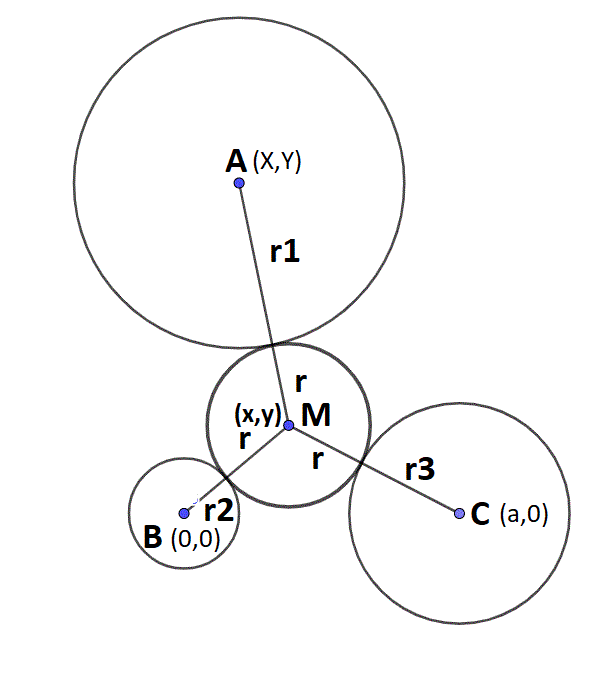


Figure S1. 2D projection of a face of tetrahedral on the xy plane

Assume a vertex B at origin (0,0), vertex C at x-axis (a, 0), and third vertex A (X, Y) can be calculated from the Euclidean distance of edges AC and AB, respectively, of the considered face (Figure S1).

In Figure S1, M represents the Maximum Circle Radius (MCR), the radius of a tangent circle that can pass through the vertices A, B, and C. The radius of MCR (r) can be easily determined by solving the subsequent set of Euclidean distance equations (detailed formulas are given in the supplementary file).

$x^{2}+y^{2}={(r_{2}+r)}^{2}$ (1)

${(x-a)}^{2}+y^{2}={(r_{3}+r)}^{2}$ (2)

${(x-X)}^{2}+{(x-Y)}^{2}={(r_{1}+r)}^{2}$ (3)

Solve equation 1 & 2 to calculate x by subtracting them

$$x=\frac{(r_{2}^{2}-r_{3}^{2}+2r_{2}r-2r_{3}r+a^{2})}{2a}$$

Simplify it by separating constant terms

$x=d_{1}r+c_{1}$ (4)

Where, $c_{1}=\frac{(r_{2}^{2}-r_{3}^{2}+a^{2})}{2a}$ , $d_{1}=\frac{(r_{2}-r_{3})}{a}$

Solve equations 1 & 3 to calculate y by subtracting them

$$y=\frac{(r_{2}^{2}-r_{1}^{2}+2r_{2}r-2r_{1}r+X^{2}+Y^{2}-2Xx)}{2Y}$$

Simplify it by separating constant terms

$y=d_{2}r+c_{2}-Px$ (5)

Where, $c_{2}=\frac{(r_{2}^{2}-r_{1}^{2}+X^{2}+Y^{2})}{2Y}$ , $d_{2}=\frac{(r_{2}-r_{1})}{Y}$, $P=\frac{X}{Y}$

Put value of x (equation 4) in the equation 5 to eliminate the x

$$y=d_{2}r+c_{2}-Pd_{1}r-Pc_{1}$$

Again, simplify it by separating constant terms

$y=d_{2}r-Rr-Q$ (6)

Where, $Q=c_{2}-Pc_{1}$, $R=Pd_{1}$

Now, to substitute the values of x and y in equation 1. The solution gives a quadratic equation of the MCR(r).

$mr^{2}+nr+s=0$ (7)

Where, $m=d_{1}^{2}+d_{2}^{2}+R^{2}-2d_{2}R-1$, $n=2(d_{2}Q+d_{1}c_{1}-RQ-r_{2})$,$s=c_{1}^{2}+Q^{2}-r_{2}^{2}$

Now, solve equation 7 to calculate the MCR that can tangentially pass through vertexes with distinct radius circles in the XY plane.

$$D=n^{2}-4ms$$

$$\boldsymbol{r=}\frac{\boldsymbol{(-n-}\sqrt{\boldsymbol{D}}\boldsymbol{)}}{\boldsymbol{2}\boldsymbol{m}}$$

**2. Pseudocode**

*BEGIN MAINPROGRAM*

*READ atom coordinates of a given protein FROM .pdb file*

*CREATE 3D Delaunay triangulation FROM atom coordinates USING the Scipy package*

*CREATE tetrahedron simplexes FROM Delaunay triangulation*

*BEGIN SUBPROGRAM to find delimiter tetrahedra of protein*

*IF -1 is the neighbor of the created tetrahedra THEN*

*Tetrahedra are on the protein surface*

*IF their circumsphere radiuses are greater than Limit THEN*

*DEFINE an ARRAY A of neighbors of these tetrahedra*

*WHILE ARRAY A is not empty*

*IF circumsphere radii of ARRAY A >Limit THEN*

*Tetrahedra are also part of the protein surface*

*DEFINE an ARRAY B of neighbors of these tetrahedra*

*ASSIGN ARRAY A = ARRAY B*

*ENDWHILE*

*Atoms present in surface tetrahedra represent surface atoms*

*Tetrahedrals originating from these atoms act as delimiter tetrahedra*

*END SUBPROGRAM to find delimiter tetrahedra of protein*

*BEGIN SUBPROGRAM to search susceptible tetrahedra*

*IF tetrahedra circumsphere radius & volume ≥ user defined Limit THEN*

*Tetrahedra are susceptible tetrahedra*

*END SUBPROGRAM to search susceptible tetrahedra*

*FOR each tetrahedral of the susceptible tetrahedra*

*DEFINE an ARRAY A for a seed tetrahedron*

*DEFINE two empty ARRAY B, C*

*//B to keep record of tetrahedra that are part of the cavity during each seed screening step*

*//C to keep record of all tetrahedra that are scanned during the seed screening step*

*WHILE ARRAY A is not empty*

*APPEND ARRAY C with ARRAY A*

*REMOVE delimiter tetrahedra from ARRAY A*

*APPEND ARRAY B with ARRAY A*

*START SUBPROGRAM to find forbidden faces for ARRAY A*

*Calculate MCR for each tetrahedron face present in ARRAY A*

*IF MCR ≤ User defined MCR THEN*

*Face is forbidden*

*Return dictionary of forbidden faces with ARRAY A as keys*

*END SUBPROGRAM to find forbidden faces for ARRAY A*

*Define new empty ARRAY D to record permitted neighbor tetrahedra*

*FOR each tetrahedron in ARRAY A*

*Remove -1 tetrahedra from its neighbors*

*Remove ARRAY C tetrahedra from its neighbors*

*Remove forbidden tetrahedra from its neighbors*

*APPEND ARRAY D with allowed neighbor tetrahedra*

*ENDFOR*

*ASSIGN ARRAY A=ARRAY D*

*ENDWHILE*

*Remove ARRAY C tetrahedra from the susceptible tetrahedra*

*FIND all atoms present at vertices of ARRAY B tetrahedra*

*IF cavity atoms ≥ Limit THEN*

*APPEND cavity count ARRAY for the given protein*

*END MAINPROGRAM*
